# The complete chloroplast genome of *Betula costata* (Betulaceae)

**DOI:** 10.1080/23802359.2020.1719928

**Published:** 2020-02-03

**Authors:** Min Woo Lee, Sang-Chul Kim, Jei-Wan Lee, Ji-Young Ahn

**Affiliations:** Division of Forest Bioinformation, National Institute of Forest Science, Suwon, Republic of Korea

**Keywords:** *Betula costata*, Betulaceae, complete chloroplast genome, phylogenetic tree

## Abstract

In the present study, we analyzed the complete chloroplast genome sequence of *Betula costata* using the Ion Torrent platform. The chloroplast genome of *B. costata* was found to be 160,547 bp in length, with a large single-copy (LSC) region of 89,385 bp, a small single-copy (SSC) region of 19,038 bp, and a pair of inverted repeats (IRs) of 26,062 bp each. The overall GC content of the chloroplast genome was 36.1%. It contained 132 genes, including 87 protein-coding genes, 8 rRNA genes, and 37 tRNA genes. The phylogenetic analysis indicated that *B. costata* is closely related to *Betula nana* and *Betula pubescens.*

The genus *Betula* (Betulaceae) is mainly distributed in the Northern Hemisphere and consists of about 60 species (Chang 2000). Five species of *Betula* (*Betula chinensis* Maxim., *Betula costata* Trautv., *Betula davurica* Pall., *Betula ermanii* Cham., and *Betula schmidtii* Regel.) are distributed in Northeast Asia. Among these, *B. costata* is distributed in the subalpine regions of Korea and Northeast China (Chen 2002; Han et al. 2012). It is used as a wooden board and its sap is used for medical purposes, such as in constipation, gout, and neuralgia (Kim et al. 1991; Choi et al. 2006). *Betula costata* was reported as one of the species used for making printing woodblocks of the Tripitaka Koreana, which was registered as a Memory of the World by UNESCO (Cho 2011). Previous studies on this species included determination of the phylogenetic relationships based on AFLP markers (Schenk et al. 2008) and sequencing of the nuclear ribosomal DNA (Li et al. 2005). However, no studies have been reported on the complete chloroplast genome sequence information. In the present study, we characterized the chloroplast DNA information in *B. costata* and analyzed its phylogenetic relationships with eight other species of Betulaceae.

Plant materials were collected from Mt. Gyebangsan, South Korea (N37°43′9.3′′, E128°26′48.5′′). Total DNA was extracted using a DNeasy Plant Mini Kit (QIAGEN, Hilden, Germany) and stored in a DNA bank (Division of Forest Bioinformation DNA bank, No. 0335133002). The whole-genome sequencing data were generated using the Ion Torrent platform (Life Technologies Corporation, Carlsbad, CA). The sequenced fragments were assembled using Geneious 10.2.3. The tRNAs were confirmed using the web-based tool, tRNAscan-SE (Lowe and Eddy 1997). A maximum-likelihood (ML) tree was constructed using the RAxML Blackbox web server, which used the rapid bootstrap analysis (Kozlov et al. 2019). The phylogenetic analysis was conducted using 100 bootstrap replicates. The complete chloroplast genome of *B. costata* (GenBank: MN830400) was found to have 160,547 bp, with a large single-copy (LSC) region of 89,385 bp, a small single-copy (SSC) region of 19,038 bp, and two inverted repeat regions (IRa and IRb) of 26,062 bp each. The overall GC content was 36.1% (LSC, 33.7%; SSC, 29.7%; IRs, 42.5%). The genome contained 132 genes, including 87 protein-coding genes, 8 rRNA genes, and 37 tRNA genes. Seven of the protein-coding genes, four rRNA genes, and seven tRNA genes, were duplicated in the IR regions. The other 15 protein-coding genes contained a single intron, and 2 genes (*ycf3* and *clpP*) had two introns. Phylogenetic analysis of *B. costata* with eight other species of *Betula*, and *Carpinus laxiflora* (using the outgroup). *Betula costata* was found to be closely related to *Betula nana* and *Betula pubescens* (Figure 1). This study provides basic information for phylogenomic studies on *B. costata* as well as on other *Betula* species.

**Figure 1.**
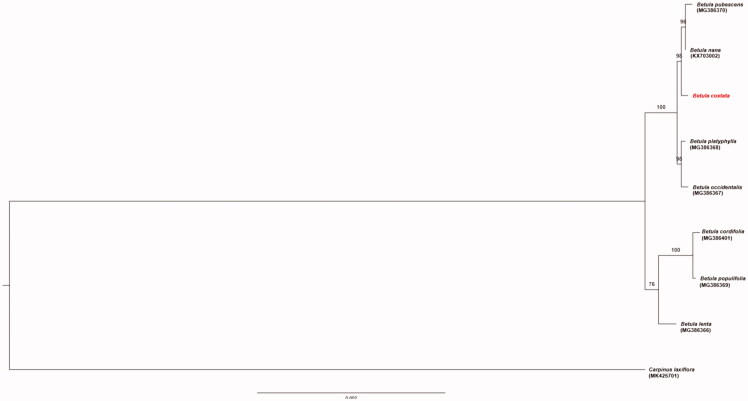
The phylogenetic tree of *B. costata* with eight species belonging to the Betulaceae based on chloroplast protein-coding sequences. Numbers in the nodes are the bootstrap values from 100 replicates.
